# Health sciences libraries’ subscriptions to journals: expectations of general practice departments and collection-based analysis

**DOI:** 10.5195/jmla.2018.282

**Published:** 2018-04-01

**Authors:** David Barreau, Céline Bouton, Vincent Renard, Jean-Pascal Fournier

**Affiliations:** Faculté de Médecine, Département de Médecine Générale, Université de Nantes, France; Faculté de Médecine, Département de Médecine Générale, Université de Nantes, France; Collège Académique, Collège National des Généralistes Enseignants, and Département de Médecine Générale, Université Paris-Est Créteil, France; Faculté de Médecine, Département de Médecine Générale, Université de Nantes, France

## Abstract

**Objective:**

The aims of this study were to (i) assess the expectations of general practice departments regarding health sciences libraries’ subscriptions to journals and (ii) describe the current general practice journal collections of health sciences libraries.

**Methods:**

A cross-sectional survey was distributed electronically to the thirty-five university general practice departments in France. General practice departments were asked to list ten journals to which they expected access via the subscriptions of their health sciences libraries. A ranked reference list of journals was then developed. Access to these journals was assessed through a survey sent to all health sciences libraries in France. Adequacy ratios (access/need) were calculated for each journal.

**Results:**

All general practice departments completed the survey. The total reference list included 44 journals. This list was heterogeneous in terms of indexation/impact factor, language of publication, and scope (e.g., patient care, research, or medical education). Among the first 10 journals listed, *La Revue Prescrire* (96.6%), *La Revue du Praticien–Médecine Générale* (90.9%), the *British Medical Journal* (85.0%), *Pédagogie Médicale* (70.0%), *Exercer* (69.7%), and the Cochrane Database of Systematic Reviews (62.5%) had the highest adequacy ratios, whereas *Family Practice* (4.2%), the *British Journal of General Practice* (16.7%), *Médecine* (29.4%), and the *European Journal of General Practice* (33.3%) had the lowest adequacy ratios.

**Conclusions:**

General practice departments have heterogeneous expectations in terms of health sciences libraries’ subscriptions to journals. It is important for librarians to understand the heterogeneity of these expectations, as well as local priorities, so that journal access meets users’ needs.

## INTRODUCTION

Health sciences libraries’ missions include subscribing to journals (print or electronic) and databases, purchasing books, and providing access to these resources through their websites [1]. One current issue for librarians is the continuous increase in costs of subscriptions to journals and databases. The average price of a scientific periodical title was $1,818 in 2016 versus $1,289 in 2011, with an annual inflation rate of 6% [2]. The selection of journal subscriptions highly depends on the budgets of libraries, which are currently declining in some countries [1, 3]. Each health sciences library is responsible for its own subscription strategy. Hence, journal subscriptions are heterogeneous among universities, and their adequacy in meeting the needs of users of health sciences libraries is unclear [4].

General practice (also known as family medicine) is defined as “an academic and scientific discipline, with its own educational content, research, evidence base and clinical activity, and a clinical specialty orientated to primary care” [5]. In France, as in many other countries, academic general practitioners are engaged in scholarly activity and, thus, may have specific requirements for access to discipline-specific journals. Previous studies have assessed the needs of general practitioners but have focused on online access, the role of practice libraries, or nonacademic general practitioners’ preferences regarding type of information sources [6–8].

The needs of academic general practitioners for journal access and the degree to which their affiliated health sciences libraries meet these needs are unknown. Also, it is uncertain whether previous literature can fit to the context of French university general practice. The authors, therefore, conducted a cross-sectional descriptive study aiming to: (i) assess the expectations of faculty in general practice departments in France regarding health sciences libraries’ subscriptions to journals and (ii) describe the current journal collections of French health sciences libraries.

## METHODS

The first phase of the study was conducted between September 17, 2015, and January 15, 2016. We contacted all of chairs and heads of scientific programs of the thirty-five university general practice departments in France via email (supplemental Appendix A). Three reminders were sent. The aims of the study were detailed in the email, and a link to an electronic questionnaire was provided (supplemental Appendix B). Participants were first asked the name of the health sciences library with which they were affiliated. The second question asked: “In order of importance, which are the ten principal journals that general practice departments should have access to via the subscriptions of health sciences libraries?” It was clearly stipulated that a response including colleagues should be given by consulting the whole team of the general practice department.

An illustrative list of fifty-seven journals was attached to the email to assist the respondents (supplemental Appendix C). This list consisted of international journals that appeared in the “Medicine, General and Internal” and “Primary Health Care” categories [9] of Journal Citation Reports (JCR) or that were familiar to us, based on our own experience. Both the questionnaire and illustrative list were pre-piloted with two French-speaking general practitioners, who were working abroad, and no major modifications were made. The questionnaire was put online using Google Forms. Responses were mandatory and collected in free-text format. For each respondent, we ranked the expectations of subscriptions to journals by attributing scores: ten for the first journal, nine for the next journal, down to one point for the last journal mentioned. The journals were then ranked by calculating the sum of all scores obtained.

The second phase of the study was conducted between January 20, 2016, and March 21, 2016, among the thirty-six existing health sciences libraries (one health sciences library per general practice department plus the interuniversity health sciences library in Paris). Based on the reference list of journals developed through the questionnaire, we asked the head librarian of each health sciences library to indicate whether a subscription for their library was available in print or electronically in 2015. The first contact with the health sciences libraries was made by phone before sending an email. Two reminders were sent. If a health sciences library did not participate despite the reminders, we collected the data using Périscope [10], which is online software that librarians use in France to compare journal collections via the catalog of the Sudoc university documentation system. All data were subsequently checked using the health sciences libraries’ websites and online catalogs. For each health sciences library, therefore, we were able to assess subscriptions to journals (print or electronic) as well as the listings of open access (OA) journal titles in the catalogs.

We counted the number of general practice departments that expected to have access to each journal on the reference list (i.e., demand) and that had access to a subscription through their health sciences library or had the journal listed in their catalog if it was an OA journal. An adequacy ratio (access/demand) was then calculated.

All data were analyzed using Microsoft Excel (version 14.0.0). Quantitative variables are represented as absolute number (percentage) unless otherwise stated. This study was conducted in agreement with the Collège National des Généralistes Enseignants (CNGE)–Collège Académique (National College of Teaching General Practitioners–Academic College). No ethics committee approval was necessary according to French law, given the observational nature of the collected data (CNGE Ethics Committee, IRB00010804, advice no. 24101727).

## RESULTS

All 35 French general practice departments participated. Forty-four journals were mentioned at least once (Table 1). Twenty-one journals (47.7%) were mentioned more than 5 times. Twelve fully OA journals (27.3%) were mentioned. The 10 principal journals to which the general practice departments expected to have access through a subscription by a health sciences library were, in decreasing order: *Exercer*, *La Revue Prescrire*, *Family Practice*, the *British Medical Journal (BMJ), Pédagogie Médicale, British Journal of General Practice, Médecine,* the *European Journal of General Practice*, *La Revue du Praticien–Médecine Générale*, and the *Cochrane Database of Systematic Reviews* (Table 2). The journals in the eleventh to forty-fourth places had scores lower than 60 and were mentioned by fewer than 12 general practice departments (Table 1).

**Table 1:**
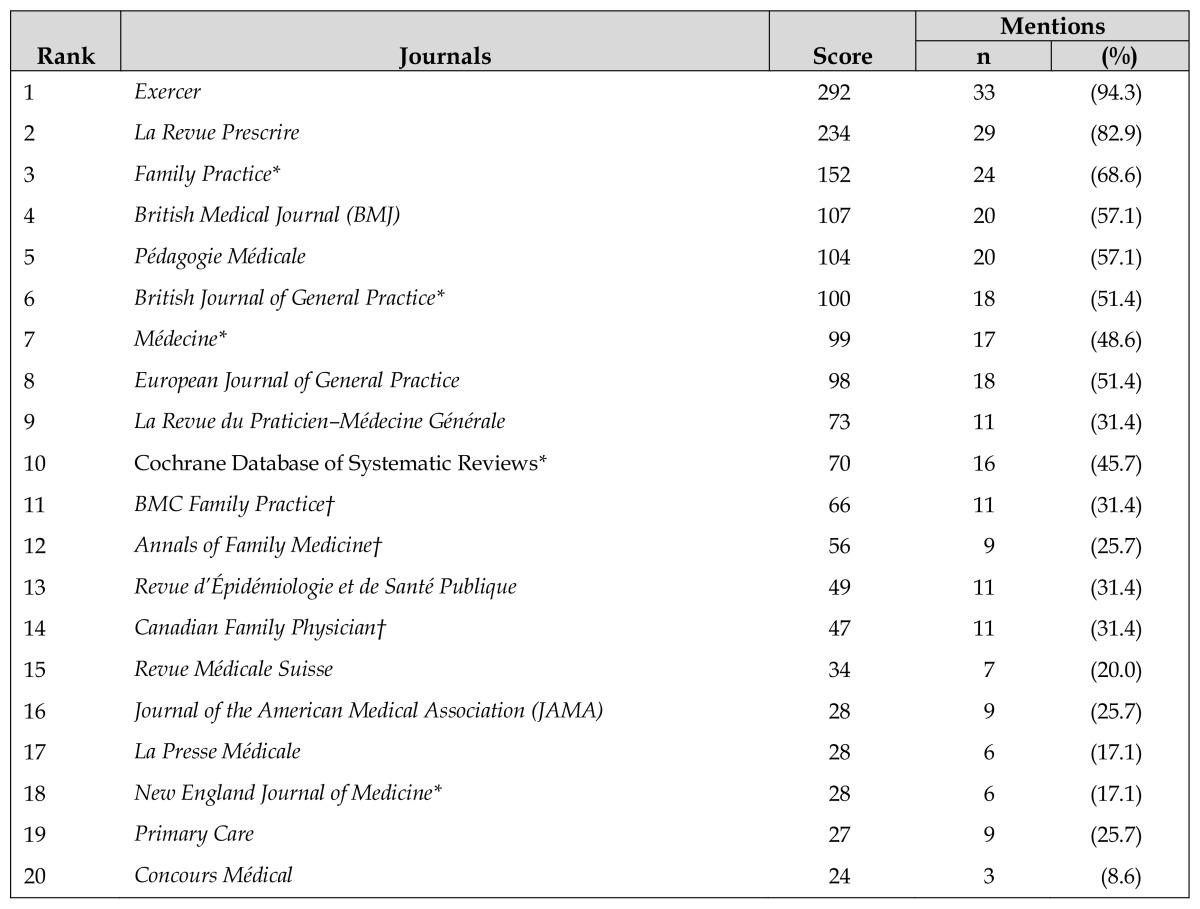
Ranked reference list of journals according to the 35 general practice departments

Rank	Journals	Score	Mentions	

			n	(%)
1	*Exercer*	292	33	(94.3)
2	*La Revue Prescrire*	234	29	(82.9)
3	Family Practice*	152	24	(68.6)
4	*British Medical Journal (BMJ)*	107	20	(57.1)
5	*Pédagogie Médicale*	104	20	(57.1)
6	British Journal of General Practice*	100	18	(51.4)
7	Médecine*	99	17	(48.6)
8	*European Journal of General Practice*	98	18	(51.4)
9	*La Revue du Praticien–Médecine Générale*	73	11	(31.4)
10	Cochrane Database of Systematic Reviews*	70	16	(45.7)
11	*BMC Family Practice*†	66	11	(31.4)
12	*Annals of Family Medicine*†	56	9	(25.7)
13	*Revue d’Épidémiologie et de Santé Publique*	49	11	(31.4)
14	*Canadian Family Physician*†	47	11	(31.4)
15	*Revue Médicale Suisse*	34	7	(20.0)
16	*Journal of the American Medical Association (JAMA)*	28	9	(25.7)
17	*La Presse Médicale*	28	6	(17.1)
18	New England Journal of Medicine*	28	6	(17.1)
19	*Primary Care*	27	9	(25.7)
20	*Concours Médical*	24	3	(8.6)
21	*Minerva Medica*	19	7	(20.0)
22	*Pratiques, les Cahiers de la Médecine Utopique*	18	6	(17.1)
23	*The Lancet*	16	4	(11.4)
24	*NPJ Primary Care Respiratory Medicine*†	15	3	(8.6)
25	*Scandinavian Journal of Primary Health Care*†	14	4	(11.4)
26	American Family Physician*	13	3	(8.6)
27	*La Revue du Praticien*	10	4	(11.4)
28	*Le Généraliste*	10	2	(5.7)
29	*JAMA Internal Medicine*	8	1	(2.9)
30	*Journal of the American Board of Family Medicine*†	8	1	(2.9)
31	*Médecine et Enfance*	8	1	(2.9)
32	*Journal of Family Practice*†	7	3	(8.6)
33	*Preventive Medicine*	6	2	(5.7)
34	*BMC Medicine*†	5	2	(5.7)
35	*PLOS Medicine*†	5	1	(2.9)
36	*Primary Care Diabetes*	5	1	(2.9)
37	*Swiss Medical Forum–Forum Médical Suisse*†	5	1	(2.9)
38	Canadian Medical Association Journal*	4	2	(5.7)
39	*Le Médecin du Québec*†	4	1	(2.9)
40	Revue Médicale de Liège*	4	1	(2.9)
41	*Australian Family Physician*†	2	1	(2.9)
42	*La Revue de Médecine Interne*	2	1	(2.9)
43	Sciences Sociales et Santé*	2	1	(2.9)
44	*Patient Education and Counseling*	1	1	(2.9)

* Open access (OA) after embargo (variable length of embargo depending on the journal).

† Fully OA.

**Table 2:** Subscriptions to the first 10 journals on the reference list for the 36 university health sciences libraries

Rank	Journal	Score	Mentions		Subscriptions						Subscrip-tion (including tax)†
		
					Print	Electronic	Print or electronic		Access*	Adequacy ratio	
	
			n	%			n	%	n	%	
1	*Exercer*	292	33	(94.3)	26	1	26	(72.2)	23	(69.7)	1,15^€^
2	*La Revue Prescrire*	234	29	(82.9)	35	15	35	(97.2)	28	(96.6)	748^€^
3	*Family Practice*	152	24	(68.6)	0	4	4	(11.1)	1	(4.2)	798^€^‡
4	*BMJ*	107	20	(57.1)	13	27	31	(86.1)	17	(85.0)	1,391^€^
5	*Pédagogie Médicale*	104	20	(57.1)	14	18	24	(66.7)	14	(70.0)	408^€^
6	*British Journal of General Practice*	100	18	(51.4)	2	7	9	(25.0)	3	(16.7)	865^€^
7	*Médecine*	99	17	(48.6)	6	8	11	(30.6)	5	(29.4)	410^€^
8	*European Journal of General Practice*	98	18	(51.4)	4	8	10	(27.8)	6	(33.3)	NA
9	*La Revue du Praticien–Médecine Générale*	73	11	(31.4)	34	27	35	(97.2)	10	(90.9)	116^€^
10	Cochrane Database of Systematic Reviews	70	16	(45.7)	NA	27	27	(75.0)	10	(62.5)	2,893^€^

* Number of libraries providing access to the journal for departments that expected access to the journal.

† Cost of a 1-year subscription for the health sciences library of the University of Nantes (France) in 2016.

‡ Tax not included.

NA: not available.

Six journals that were mentioned did not appear in the representative list, and each was mentioned fewer than 2 times: *Le Généraliste*, *Médecine et Enfance*, *Swiss Médical Forum–Forum Medical Suisse*, *Le Médecin du Québec*, *La Revue Médicale de Liège*, and *Sciences Sociales et Sant*. Of the 44 journals mentioned, 16 (36.4%) were in French, 3 (6.8%) were bilingual (*Swiss Medical Forum*, *Le Médecin du Québec*, *Canadian Family Physician*), and the others (56.8%) were in English. Twenty-eight journals (63.6%) had an impact factor (IF) in JCR.

Of the 36 health sciences libraries contacted, 35 returned the list of their collections (97.2%). We used Périscope data for 1 health sciences library. The complete health sciences library offerings for each of the 44 journals are presented in supplemental Table 3. The fully OA journals were not listed in all of the library catalogs. Thirty-two journals (72.7%) were provided more frequently in electronic format than in print format. Table 2 shows the subscription offerings to the first 10 journals on the reference list.

Supplemental Table 4 shows the demand, access, and corresponding adequacy ratios for all 44 journals on the reference list. An adequacy ratio greater than 50% was obtained for 27 journals (61.4%). Table 2 summarizes adequacy for the first 10 journals. Of these, the 3 journals with the highest adequacy ratios were *La Revue Prescrire*, *La Revue du Praticien-Médecine Générale*, and *BMJ*, and the 3 journals with the lowest adequacy ratios were *Family Practice*, the *British Journal of General Practice*, and *Médecine*. An adequacy ratio greater than 50% was obtained for 6 of these 10 journals (60%).

## DISCUSSION

We identified and prioritized forty-four journals, based on expectations of library subscriptions of the thirty-five French general practice departments. The journal subscription offerings of the thirty-six French health sciences libraries were heterogeneous, and adequacy in meeting demand was imperfect. Among the first ten journals listed, *La Revue Prescrire*, *La Revue du Praticien–Médecine Générale*, *BMJ*, *Pédagogie Médicale*, *Exercer,* and the Cochrane Database of Systematic Reviews had the best adequacy, whereas *Family Practice*, the *British Journal of General Practice*, *Médecine,* and the *European Journal of General Practice* had the least adequacy.

### Expectations of general practice departments

Great heterogeneity was noted among lists of journals mentioned by different departments in terms of scores, number of mentions, language of publication, IF, and scope. Cross-checking this list to previously developed lists (supplemental Table 5) illuminates the diversity of journals related to general practice. For example, the World Organization of National Colleges, Academies and Academic Associations of General Practitioners/Family Physicians (WONCA) provides six lists of up to sixty-five “journals of interest” for general practitioners [11]. In particular, WONCA’s lists of thirty-one general practice journals in English, fourteen general practice journals in other languages, and ten general internal medicine journals contain ten, two, and five journals identified in the present study, respectively.

Similar observations were made with the 2003 Brandon/Hill list [12], the McKibbon et al. list [8], and the Alper et al. list [13]. This limited overlap between purportedly “core lists” of general practice journals has been highlighted in a previous study aiming to identify relevant literature for primary care physicians [13]. This heterogeneity might be due to differences in the methods used for list creation, the context of the lists (e.g., country), or their target audiences (e.g., clinician, researcher, educator). Our study results suggest that relying on users’ expectations may be a complementary way to produce a comprehensive list of general practice journals.

The journal mentioned most frequently in our reference list was *Exercer*. This journal provides consensus in the French general practice community [14], although it is not indexed in MEDLINE and has no IF in JCR. This journal is developed and published by the CNGE, and because this study was conducted in partnership with the CNGE–Collège Académique, it is possible that the respondents were influenced to name this journal.

*La Revue Prescrire* was in second place on the list. Like *Exercer*, it is not indexed in MEDLINE and has no IF. This journal is more oriented toward therapeutics than the general practice discipline. Its high ranking might be related to its independence from industrial interests. It is the second journal (after *Exercer*) in terms of numbers of individual subscriptions among French teaching general practitioners (85.2%) [14].

In third place was the international journal *Family Practice*. This is the first journal on the reference list that is indexed in MEDLINE. Nevertheless, it has a low IF (2.0 in 2015), compared to other journals with lower scores.

Journals in the fourth through tenth positions had similar scores, and almost one-third of general practice departments expected to have access to them. International journals with a high IF, such as *BMJ* (IF of 19.7 in 2015), or a lower, IF such as the *British Journal of General Practice* (IF of 2.7 in 2015) and the *European Journal of General Practice* (IF of 1.4 in 2015), had the same degree of expectation as French non-indexed journals such as *Pédagogie Médicale*, *Médecine,* and *La Revue du Praticien–Médecine Générale*.

From the eleventh position onward, expectations were lower but still heterogeneous. These journals included some internationally highly reputed journals, such as the *New England Journal of Medicine* (IF of 59.6 in 2015), *The Lancet* (IF at 44.0 in 2015), and *JAMA* (IF at 37.7 in 2015), which had IFs far superior to those of the first journals listed. Overall, therefore, IF does not appear to be a major factor influencing expectations of general practice departments.

A recent survey identified the multiple expectations of teaching general practitioners with regard to what a general practice journal should address [14]. The most expected themes were care, research, and medical education, respectively. Indeed, the first ten journals on the list reflected the diversity of these expectations. Although some journals were exclusively oriented toward care (e.g., *La Revue du Praticien–Médecine Générale*) and others were only oriented toward medical education (e.g., *Pédagogie Médicale*), most addressed all three themes and, thus, are important to both teachers and researchers in general practice.

### Imperfect adequacy between the demand for journals by general practice departments and their access through health sciences libraries

Among the first 10 journals on the list, high degrees of adequacy were observed for *La Revue Prescrire*, *La Revue du Praticien–Médecine Générale,* and *BMJ* (96.9%, 90.9%, and 85.0%, respectively). *Pédagogie Médicale* and *Exercer* had acceptable scores of around 70.0%. Conversely, *Family Practice*, the *British Journal of General Practice,* and *Médecine* had low adequacy scores (4.2%, 16.7%, and 29.4%, respectively), although their subscription costs were similar to those of the other journals (Table 2). One can surmise that these 3 journals were rarely purchased by health sciences libraries because their contents are made OA after a 12-month embargo period.

When purchasing and cancelling journal subscriptions, librarians consider the costs of the subscriptions, the existence of licenses, the possibility of OA after a period of embargo, the possibility of access to secondary sources, and usage metrics [15]. The journal subscription offerings of health sciences libraries are largely influenced by increasing subscription costs in the face of constrained budgets [1]. Some health sciences libraries have been forced to cancel subscriptions to journals despite being used and endorsed by health sciences library users. This is the case of the interuniversity health science library in Paris that, in view of its age, used to house the largest collection of journals of all French universities. This library pays two- to three-fold more for journal subscriptions than comparable institutions. It was forced to cancel more than 650 titles between 2007 and 2013, while retaining journals in the collection deals to which it had previously subscribed [16]. The health sciences library of Toulouse informed us that, in 2016, it had cancelled subscriptions to *Family Practice*, the *European Journal of General Practice,* and the *British Journal of General Practice* for similar reasons.

### Strengths and weaknesses of the present study

To our knowledge, this is the first study investigating the expectations of general practice departments regarding journal subscriptions by health sciences libraries. We obtained exhaustive and collegial participation of general practice departments and nearly all health sciences libraries in France.

However, some limitations should be acknowledged. First, the illustrative list of journals that accompanied the questionnaire might have influenced the respondents. To date, there is no consensual French reference list of journals dedicated to general practice, and we considered this list necessary to assist the respondents. Second, confusion in the names of the journals might have existed. For example, *Minerva Medica* may have been confused with the Belgian journal of evidence-based medicine, *Minerva*. Third, we only took into account the subscriptions of health sciences libraries as a mechanism of access to up-to-date medical information. Alternative access strategies should be kept in mind for a cautious appraisal of study results. For example, some general practice departments or teachers and researchers might subscribe to journals individually [14]. Others might use subscriptions of other organizations with which they are affiliated (e.g., institutes, hospitals) or share access codes between teachers and researchers. The rapid extension of pirating or sharing of articles on community websites [17] might also impact the expectations of users of health sciences libraries. Fourth, adequacy ratios for OA journals should, in theory, have been 100%. However, some OA journals were not listed in the health sciences libraries’ collections, leading to lower adequacy rates. Last, this study focused only on the expectations of users of health sciences libraries. A supplemental assessment of actual usage (i.e., journals borrowed, interlibrary loans, article downloads) is required to gain precise information on the needs of users and to optimize the offerings of general practice journals by health sciences libraries [18–20].

### Areas for improvement

On a national level, the sharing of resources is necessary to obtain better offerings of journals at the best costs. In France, Couperin (unified consortium of the university and research establishments for the access to digital publications) is a nonprofit organization financed by the contributions of its members and government grants (Ministry of Higher Education and Research) [21]. This consortium serves as a network for negotiations with publishers and provides expertise in digital information resources for research and higher education in France. Today, Couperin has more than 250 members, more than half of which are universities.

On a local level, sharing electronic collections between institutions avoids the uncoordinated dispersion of purchases. For example, agreements between university hospital centers and health sciences libraries have avoided duplicate subscriptions [4, 22]. Furthermore, organized pay-per-view purchase or loan of articles by health sciences libraries can help decrease subscription costs and provide personalized offerings to library users [3]. Lastly, reinforced collaboration between university librarians and users is required. Presently, health sciences libraries are still perceived in France as an authority that refuses to purchase new subscriptions [22]. Abroad, experiences of reinforced collaboration between general practitioners and librarians have shown their efficacy in adapting health sciences libraries’ offerings to general practitioners’ requirements [6]. With the construction of a reference list of journals and the results of the present study, we hope that each general practice department will be able to discuss journal subscriptions with its health sciences library so that library offerings more closely align with academic general practitioner needs.

## SUPPLEMENTAL FILES

Appendix A Invitation email

Appendix B Online questionnaire

Appendix C Illustrative list of fifty-seven journals

Table 3 Subscription offerings to the 44 journals on the reference list for the 36 university health sciences libraries in 2015

Table 4 Adequacy between the demand for journals by general practice departments and their access through health sciences libraries in 2015

Table 5 Cross-checking between the ranked list of 44 journals identified by university general practice departments and previously developed lists
