# Supplementary material for: Health sciences libraries’ subscriptions to journals: expectations of general practice departments and collection-based analysis
Source: J Med Libr Assoc. 2018 Apr 1;106(2):235–43. doi: 10.5195/jmla.2018.282 (PMC5886506; doi:10.5195/jmla.2018.282)
Supplement: Appendix A [file jmla-106-235-s001.pdf]

JMLA Journal of the Medical Library Association

SC
Supplemental content to
J Med Libr Assoc. Apr;106(2):dx.doi.org/10.5195/jmla.2018.282
www.jmla.mlanet.org
© Barreau, Bouton, Renard, Fournier 2018

Health sciences libraries' subscriptions to journals: expectations of general practice departments and collection-based analysis

David Barreau; Céline Bouton; Vincent Renard; Jean-Pascal Fournier

APPENDIX A

Invitation email

CNGE COLLÈGE ACADÉMIQUE — DMG NANTES — UNIVERSITÉ DE NANTES

Objet : participation à une étude sur les abonnements des BU Santé aux revues de la discipline médecine générale

Nantes, le 14 septembre 2015

Bonjour,

Le Département de Médecine Générale (DMG) de Nantes réalise une étude pour évaluer les attentes des DMG en termes d'abonnements des bibliothèques universitaires (BU) santé aux revues de notre discipline.

Ce projet est soutenu et mené en accord avec le CNGE Collège académique.

Nous souhaitons réaliser un **sondage** au sein de votre DMG, afin de lister (par ordre d'importance) les dix revues auxquelles votre équipe estime que les DMG devraient avoir accès via les abonnements des BU Santé. Pour cela, nous avons invité **le référent recherche de votre département à centraliser la réponse de votre équipe** (il a reçu dans un mail personnel le lien pour remplir le questionnaire).

Aucune liste consensuelle de revue de la discipline « médecine générale » n'existe à ce jour. Pour vous aider, vous trouverez ci-joint un document avec une liste indicative, mais non limitative, de revues pouvant entrer dans le champ de notre discipline.

En retour, nous nous engageons à vous fournir les données de l'étude qui vous permettront, nous l'espérons, de renégocier les abonnements de votre BU santé, afin qu'ils soient au plus près des besoins de votre équipe.

Cette étude fera l'objet de la thèse d'exercice de David Barreau, interne en Médecine générale à Nantes, sous la direction du Dr Jean-Pascal Fournier.

Nous vous remercions très sincèrement pour votre aide et restons à votre disposition pour toute demande complémentaire.

Cordialement,

Jean-Pascal Fournier (CCU-MG, DMG Nantes)
jean-pascal.fournier@univ-nantes.fr

David Barreau (IMG, DMG Nantes)
barreau.david@hotmail.com,
+33 6 10 46 44 61
