# Supplementary material for: Health sciences libraries’ subscriptions to journals: expectations of general practice departments and collection-based analysis
Source: J Med Libr Assoc. 2018 Apr 1;106(2):235–43. doi: 10.5195/jmla.2018.282 (PMC5886506; doi:10.5195/jmla.2018.282)
Supplement: Appendix B [file jmla-106-235-s002.pdf]

Supplemental content to
J Med Libr Assoc. Apr;106(2):dx.doi.org/10.5195/jmla.2018.282
www.jmla.mlanet.org
© Barreau, Bouton, Renard, Fournier 2018

Health sciences libraries' subscriptions to journals: expectations of general practice departments and collection-based analysis

David Barreau; Céline Bouton; Vincent Renard; Jean-Pascal Fournier

APPENDIX B

Online questionnaire

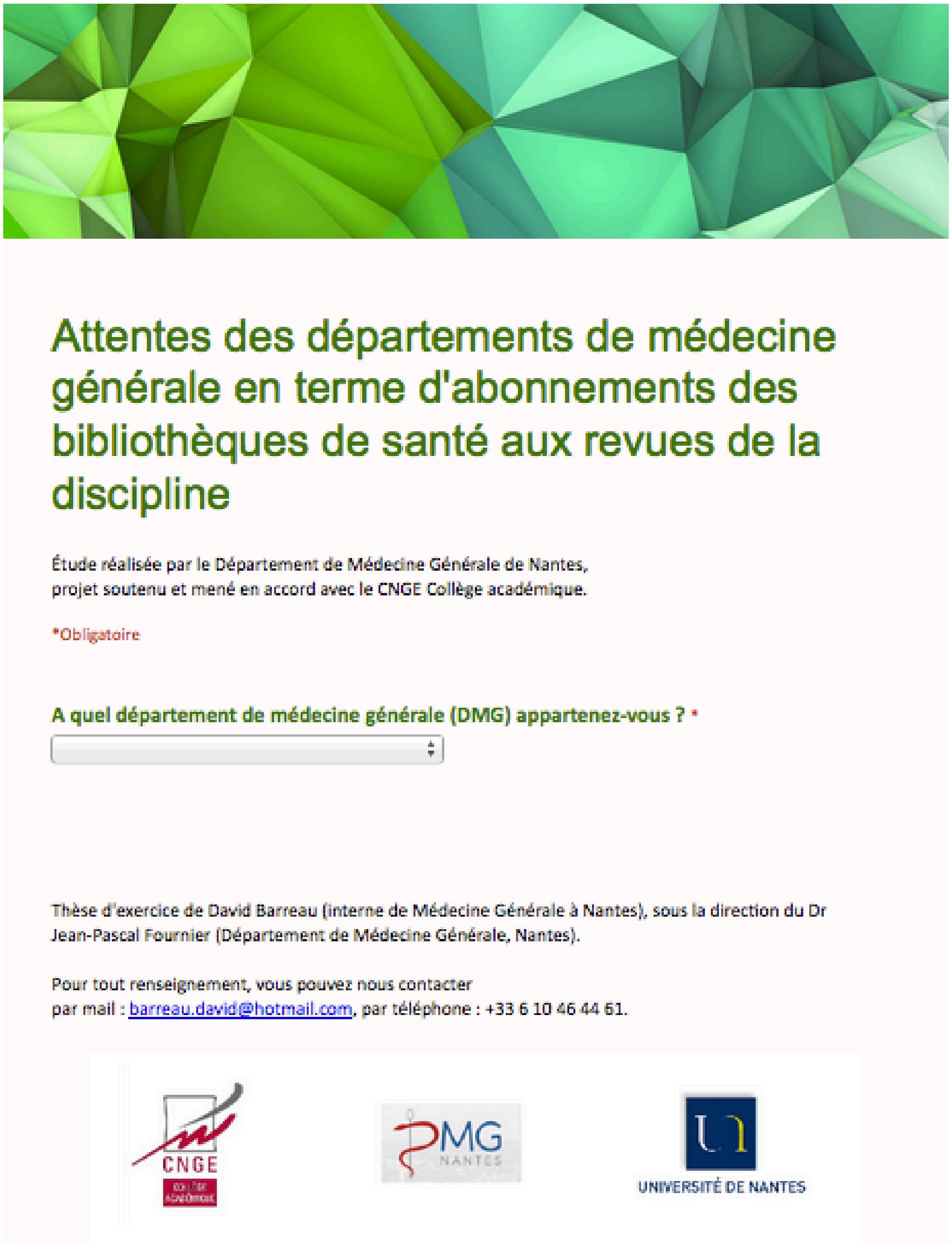

Attentes des départements de médecine générale en terme d'abonnements des bibliothèques de santé aux revues de la discipline

Étude réalisée par le Département de Médecine Générale de Nantes,
projet soutenu et mené en accord avec le CNGE Collège académique.

*Obligatoire

A quel département de médecine générale (DMG) appartenez-vous ? *

Thèse d'exercice de David Barreau (interne de Médecine Générale à Nantes), sous la direction du Dr Jean-Pascal Fournier (Département de Médecine Générale, Nantes).

Pour tout renseignement, vous pouvez nous contacter
par mail : barreau.david@hotmail.com, par téléphone : +33 6 10 46 44 61.

Journal of the Medical Library Association

Supplemental content to
J Med Libr Assoc. Apr;106(2):dx.doi.org/10.5195/jmla.2018.282
www.jmla.mlanet.org
© Barreau, Bouton, Renard, Fournier 2018

Attentes des départements de médecine générale en terme d'abonnements des bibliothèques de santé aux revues de la discipline

Étude réalisée par le Département de Médecine Générale de Nantes,
projet soutenu et mené en accord avec le CNGE Collège académique.

*Obligatoire

A quel département de médecine générale (DMG) appartenez-vous ? *

- Aix-Marseille
- Amiens
- Angers
- Antilles-Guyane
- Besançon
- Bordeaux
- Brest
- Caen
- Clermont-Ferrand
- Corse
- Dijon
- Grenoble
- La Réunion - Océan Indien
- Lille
- Lille Catho
- Limoges
- Lyon
- Montpellier
- Nancy

lecine Générale à Nantes), sous la direction du Dr
énérale, Nantes).

cter
one : +33 6 10 46 44 61.

MG
ANTES

UNIVERSITÉ DE NANTES

SC
Supplemental content to
J Med Libr Assoc. Apr;106(2):dx.doi.org/10.5195/jmla.2018.282
www.jmla.mlanet.org
© Barreau, Bouton, Renard, Fournier 2018

Attentes des départements de médecine générale en terme d'abonnements des bibliothèques de santé aux revues de la discipline

*Obligatoire

Étude réalisée par le Département de Médecine Générale de Nantes,
projet soutenu et mené en accord avec le CNGE Collège académique.

Quelle(s) est (sont) la (les) bibliothéque(s) universitaire(s) de santé rattachée(s) à votre département? * *

Thèse d'exercice de David Barreau (interne de Médecine Générale à Nantes), sous la direction du Dr Jean-Pascal Fournier (Département de Médecine Générale, Nantes).

Pour tout renseignement, vous pouvez nous contacter
par mail : barreau.david@hotmail.com, par téléphone : +33 6 10 46 44 61.

CNGE COLLÈGE ACADÉMIQUE — DMG NANTES — UNIVERSITÉ DE NANTES

« Retour | Continuer »

Terminé à 50 %

Supplemental content to
J Med Libr Assoc. Apr;106(2):dx.doi.org/10.5195/jmla.2018.282
www.jmla.mlanet.org
© Barreau, Bouton, Renard, Fournier 2018

Attentes des départements de médecine générale en terme d'abonnements des bibliothèques de santé aux revues de la discipline

*Obligatoire

Étude réalisée par le Département de Médecine Générale de Nantes,
projet soutenu et mené en accord avec le CNGE Collège académique.

En vous appuyant sur votre équipe pour répondre :

Quelles sont, par ordre d'importance, les 10 principales revues de la discipline médecine générale, auxquelles les départements de médecine générale devraient avoir accès via les abonnements des bibliothèques universitaires ?

Lien vers une liste non limitative proposée à titre indicatif :

https://docs.google.com/drawings/d/1CHecknAsf_DKRrwuLA-Q7lrSa7rDh1KnrKOMKoJrfDU/edit?usp=sharing

La revue de la discipline n°1 *

La revue de la discipline n°2 *

La revue de la discipline n°3 *

La revue de la discipline n°4 *

SC
Supplemental content to
J Med Libr Assoc. Apr;106(2):dx.doi.org/10.5195/jmla.2018.282
www.jmla.mlanet.org
© Barreau, Bouton, Renard, Fournier 2018

La revue de la discipline n°5 *

La revue de la discipline n°6 *

La revue de la discipline n°7 *

La revue de la discipline n°8 *

La revue de la discipline n°9 *

La revue de la discipline n°10 *

Thèse d'exercice de David Barreau (interne de Médecine Générale à Nantes), sous la direction du Dr Jean-Pascal Fournier (Département de Médecine Générale, Nantes).

Pour tout renseignement, vous pouvez nous contacter
par mail : barreau.david@hotmail.com, par téléphone : +33 6 10 46 44 61.

CNGE COLLÈGE ACADÉMIQUE — DMG NANTES — UNIVERSITÉ DE NANTES

« Retour Continuer »

Terminé à 75 %

JMLA Journal of the Medical Library Association

SC
Supplemental content to
J Med Libr Assoc. Apr;106(2):dx.doi.org/10.5195/jmla.2018.282
www.jmla.mlanet.org
© Barreau, Bouton, Renard, Fournier 2018

Attentes des départements de médecine générale en terme d'abonnements des bibliothèques de santé aux revues de la discipline

*Obligatoire

Afin de recevoir les résultats de cette étude, merci de nous communiquer le nom et l'adresse email du référent recherche qui a centralisé la réponse de votre équipe.

Nom du référent : *

(un seul interlocuteur par DMG)

Mail du référent : *

Thèse d'exercice de David Barreau (interne de Médecine Générale à Nantes), sous la direction du Dr Jean-Pascal Fournier (Département de Médecine Générale, Nantes).

Pour tout renseignement, vous pouvez nous contacter
par mail : barreau.david@hotmail.com, par téléphone : +33 6 10 46 44 61.

CNGE COLLÈGE ACADÉMIQUE — DMG NANTES — UNIVERSITÉ DE NANTES

« Retour | Envoyer

N'envoyez jamais de mots de passe via Google Forms.

100 % : vous avez réussi.

SC
Supplemental content to
J Med Libr Assoc. Apr;106(2):dx.doi.org/10.5195/jmla.2018.282
www.jmla.mlanet.org
© Barreau, Bouton, Renard, Fournier 2018

Attentes des départements de médecine générale en terme d'abonnements des bibliothèques de santé aux revues de la discipline

Votre réponse a bien été enregistrée.

Merci pour votre collaboration. Nous espérons que cette étude aidera à améliorer vos accès à ces revues de la discipline.

Thèse d'exercice de David Barreau (interne de Médecine Générale à Nantes), sous la direction du Dr Jean-Pascal Fournier (Département de Médecine Générale, Nantes).

Pour tout renseignement, vous pouvez nous contacter
par mail : barreau.david@hotmail.com, par téléphone : +33 6 10 46 44 61.

Ce formulaire a été créé à l'aide de Google Forms.
Créer votre formulaire

Google Forms
