# Supplementary material for: Health sciences libraries’ subscriptions to journals: expectations of general practice departments and collection-based analysis
Source: J Med Libr Assoc. 2018 Apr 1;106(2):235–43. doi: 10.5195/jmla.2018.282 (PMC5886506; doi:10.5195/jmla.2018.282)
Supplement: Appendix C [file jmla-106-235-s003.pdf]

JMLA Journal of the Medical Library Association

SC

Supplemental content to
J Med Libr Assoc. Apr;106(2):dx.doi.org/10.5195/jmla.2018.282
www.jmla.mlanet.org
© Barreau, Bouton, Renard, Fournier 2018

Health sciences libraries' subscriptions to journals: expectations of general practice departments and collection-based analysis

David Barreau; Céline Bouton; Vincent Renard; Jean-Pascal Fournier

APPENDIX C

Illustrative list of fifty-seven journals

CNGE COLLÈGE ACADÉMIQUE — DMG NANTES — UNIVERSITÉ DE NANTES

Liste non exhaustive de revues pouvant entrer dans le champ de la médecine générale

- American Family Physician
- Annals of Internal Medicine
- Annals of Medicine
- Archives of Internal Medicine
- Atención Primaria
- Australian Family Physician
- Australian Journal of Primary Health
- BMC Family Practice
- BMC Medicine
- British Medical Journal
- British Medical Journal Open
- Bulletin de l'Académie Nationale de Médecine
- Canadian Family Physician
- Canadian Medical Association Journal
- Concours Médical (Le)
- European Journal of General Practice
- Exercer
- Family Medicine
- Family Practice
- Impact Médecine
- International Journal of Clinical Practice
- JAMA Internal Medicine
- JAMA Journal of the American Medical Association
- Journal of the American Board of Family Medicine
- La Presse Médicale
- La Revue de Médecine Interne
- La Revue du Praticien
- La Revue du Praticien - Médecine Générale
- Mayo Clinic Proceedings
- Médecin généraliste (Le)
- Médecine
- Minerva Medica
- Pédagogie médicale
- PLOS Medicine
- Pratiques, les cahiers de la médecine utopique
- Pratiques et organisation des soins
- Preventive Medicine
- Primary Care
- Primary Care Diabetes
- Primary Care Respiratory Journal
- QJM: An International Journal of Medicine
- Revue d'Epidémiologie et de Santé Publique
- Revue Médicale Suisse
- Revue Prescrire
- Scandinavian Journal of Primary Health Care
- The American Journal of Medicine
- The Annals of Family Medicine
- The British Journal of General Practice
- The Cochrane Database of Systematic Reviews
- The Journal of Family Practice
- The Journal of General Internal Medicine
- The Journal of the American Board of Family Practice
- The Lancet
- The Medical Journal of Australia
- The New England Journal of Medicine
- The Physician and Sportsmedicine
- The Swiss Medical Weekly
