# Supplementary material for: Health sciences libraries’ subscriptions to journals: expectations of general practice departments and collection-based analysis
Source: J Med Libr Assoc. 2018 Apr 1;106(2):235–43. doi: 10.5195/jmla.2018.282 (PMC5886506; doi:10.5195/jmla.2018.282)
Supplement: Table 3 [file jmla-106-235-s004.pdf]

JMLA Journal of the Medical Library Association

SC

Supplemental content to
J Med Libr Assoc. Apr;106(2):dx.doi.org/10.5195/jmla.2018.282
www.jmla.mlanet.org
© Barreau, Bouton, Renard, Fournier 2018

Health sciences libraries' subscriptions to journals: expectations of general practice departments and collection-based analysis

David Barreau; Céline Bouton; Vincent Renard; Jean-Pascal Fournier

Table 3

Subscription offerings to the 44 journals on the reference list for the 36 university health sciences libraries in 2015

Journal	Subscriptions			
			Print or electronic	
	Print	Electronic	n	(%)
*American Family Physician**	1	1	1	(2.8)
Annals of Family Medicine†	0	32	32	(88.9)
Australian Family Physician†	0	16	16	(44.4)
BMC Family Practice†	NA	32	32	(88.9)
BMC Medicine†	NA	31	31	(86.1)
British Medical Journal (BMJ)	13	27	31	(86.1)
*British Journal of General Practice**	2	7	9	(25.0)
Canadian Family Physician†	1	31	31	(86.1)
*Canadian Medical Association Journal**	1	1	2	(5.6)
Cochrane Database of Systematic Reviews*	NA	27	27	(75.0)
Concours Médical	30	22	31	(86.1)
European Journal of General Practice	4	8	10	(27.8)
Exercer	26	1	26	(72.2)
*Family Practice**	0	4	4	(11.1)
Journal of the American Medical Association (JAMA)	21	15	29	(80.6)
JAMA Internal Medicine	16	9	22	(61.1)
Journal of the American Board of Family Medicine†	1	25	26	(72.2)
La Presse Médicale	11	27	31	(86.1)
La Revue de Médecine Interne	2	29	30	(83.3)
La Revue du Praticien	35	29	36	(100.0)
La Revue du Praticien–Médecine Générale	34	27	35	(97.2)
La revue Prescrire	35	15	35	(97.2)
Le Généraliste	11	3	13	(36.1)
Le Médecin du Québec†	0	10	10	(27.8)
*Médecine**	6	8	11	(30.6)
Médecine et Enfance	7	2	8	(22.2)
Minerva Medica	0	2	2	(5.6)
NPJ Primary Care Respiratory Medicine†	0	29	29	(80.6)
Patient Education and Counseling	2	30	30	(83.3)
Pédagogie Médicale	14	18	24	(66.7)
PLOS Medicine†	NA	33	33	(91.7)
Pratiques. les Cahiers de la Médecine Utopique	15	5	18	(50.0)

© Barreau, Bouton, Renard, Fournier 2018

Journal	Subscriptions			
	Print	Electronic	Print or electronic	
			n	(%)
Preventive Medicine	1	31	31	(86.1)
Primary Care	0	10	10	(27.8)
Primary Care Diabetes	1	32	32	(88.9)
Revue d'Épidémiologie et de Santé Publique	8	26	28	(77.8)
*Revue Médicale de Liège**	5	2	6	(16.7)
Revue Médicale Suisse	16	5	17	(47.2)
Scandinavian Journal of Primary Health Care†	0	18	18	(50.0)
Sciences Sociales et Santé	7	17	19	(52.8)
Swiss Medical Forum–Forum Médical Suisse†	0	24	24	(66.7)
Journal of Family Practice†	2	23	24	(66.7)
The Lancet	3	31	31	(86.1)
*New England Journal of Medicine**	19	23	33	(91.7)

* Open access (OA) after embargo (variable length of embargo depending on the journal).
† Fully OA.
NA: Not applicable (absence of paper format).
