# Supplementary material for: Health sciences libraries’ subscriptions to journals: expectations of general practice departments and collection-based analysis
Source: J Med Libr Assoc. 2018 Apr 1;106(2):235–43. doi: 10.5195/jmla.2018.282 (PMC5886506; doi:10.5195/jmla.2018.282)
Supplement: Table 4 [file jmla-106-235-s005.pdf]

JMLA Journal of the Medical Library Association

SC

Supplemental content to
J Med Libr Assoc. Apr;106(2):dx.doi.org/10.5195/jmla.2018.282
www.jmla.mlanet.org
© Barreau, Bouton, Renard, Fournier 2018

# Health sciences libraries' subscriptions to journals: expectations of general practice departments and collection-based analysis

**David Barreau; Céline Bouton; Vincent Renard; Jean-Pascal Fournier**

**Table 4**

**Adequacy between the demand for journals by general practice departments and their access through health sciences libraries in 2015**

| Journal | Demand | Access* | Adequacy ratio (%) |
|---|---|---|---|
| *American Family Physician†* | 3 | 0 | – |
| *Annals of Family Medicine‡* | 9 | 8 | 88.9 |
| *Australian Family Physician‡* | 1 | 0 | – |
| *BMC Family Practice‡* | 11 | 7 | 63.6 |
| *BMC Medicine‡* | 1 | 1 | 100.0 |
| *British Medical Journal (BMJ)* | 20 | 17 | 85.0 |
| *British Journal of General Practice†* | 18 | 3 | 16.7 |
| *Canadian Family Physician‡* | 11 | 9 | 81.8 |
| *Canadian Medical Association Journal†* | 2 | 0 | – |
| Cochrane Database of Systematic Reviews | 16 | 10 | 62.5 |
| *Concours Médical* | 3 | 2 | 66.7 |
| *European Journal of General Practice* | 18 | 6 | 33.3 |
| *Exercer* | 33 | 23 | 69.7 |
| *Family Practice†* | 24 | 1 | 4.2 |
| *Journal of the American Medical Association (JAMA)* | 6 | 5 | 83.3 |
| *JAMA Internal Medicine* | 1 | 1 | 100.0 |
| *Journal of the American Board of Family Medicine‡* | 1 | 1 | 100.0 |
| *La Presse Médicale* | 6 | 5 | 83.3 |
| *La Revue de Médecine Interne* | 1 | 1 | 100.0 |
| *La Revue du Praticien* | 4 | 4 | 100.0 |
| *La Revue du Praticien–Médecine Générale* | 11 | 10 | 90.9 |
| *La Revue Prescrire* | 29 | 28 | 96.6 |
| *Le Généraliste* | 2 | 1 | 50.0 |
| *Le Médecin du Québec‡* | 1 | 0 | – |
| *Médecine* | 17 | 5 | 29.4 |
| *Médecine et Enfance* | 1 | 0 | – |
| *Minerva Medica* | 7 | 0 | – |
| *NPJ Primary Care Respiratory Medicine‡* | 3 | 2 | 66.7 |
| *Patient Education and Counseling* | 1 | 1 | 100.0 |
| *Pédagogie Médicale* | 20 | 14 | 70.0 |
| *PLOS Medicine‡* | 1 | 1 | 100.0 |
| *Pratiques, les Cahiers de la Médecine Utopique* | 6 | 5 | 83.3 |

JMLA Journal of the Medical Library Association

SC

Supplemental content to
J Med Libr Assoc. Apr;106(2):dx.doi.org/10.5195/jmla.2018.282
www.jmla.mlanet.org
© Barreau, Bouton, Renard, Fournier 2018

| Journal | Demand | Access* | Adequacy ratio (%) |
|---|---|---|---|
| *Preventive Medicine* | 2 | 2 | 100.0 |
| *Primary Care* | 9 | 2 | 22.2 |
| *Primary Care Diabetes* | 2 | 1 | 50.0 |
| *Revue d'Épidémiologie et de Santé Publique* | 11 | 9 | 81.8 |
| *Revue Médicale de Liège*† | 1 | 0 | – |
| *Revue Médicale Suisse* | 7 | 2 | 28.6 |
| *Scandinavian Journal of Primary Health Care*‡ | 4 | 1 | 25.0 |
| *Sciences Sociales et Santé*† | 1 | 0 | – |
| *Swiss Medical Forum–Forum Médical Suisse*‡ | 1 | 0 | – |
| *Journal of Family Practice*‡ | 3 | 1 | 33.3 |
| *The Lancet* | 4 | 3 | 75.0 |
| *New England Journal of Medicine* | 9 | 9 | 100.0 |

* Number of libraries providing access to the journal for departments that expected access to the journal.

† Open access (OA) after embargo (variable length of embargo depending on the journal).

‡ Fully OA.
