# Supplementary material for: Health sciences libraries’ subscriptions to journals: expectations of general practice departments and collection-based analysis
Source: J Med Libr Assoc. 2018 Apr 1;106(2):235–43. doi: 10.5195/jmla.2018.282 (PMC5886506; doi:10.5195/jmla.2018.282)
Supplement: Table 5 [file jmla-106-235-s006.pdf]

JMLA Journal of the Medical Library Association

SC

Supplemental content to
J Med Libr Assoc. Apr;106(2):dx.doi.org/10.5195/jmla.2018.282
www.jmla.mlanet.org
© Barreau, Bouton, Renard, Fournier 2018

Health sciences libraries' subscriptions to journals: expectations of general practice departments and collection-based analysis

David Barreau; Céline Bouton; Vincent Renard; Jean-Pascal Fournier

Table 5

Cross-checking between the ranked list of 44 journals identified by university general practice departments and previously developed lists

	Journals	WONCA [1]			Brandon/Hill (2003) [2]		McKibbon (2007) [3]	Alper (2004) [4]
		General practice (English)	General practice (non-English)	General/Internal medicine	General practice	General/Internal medicine		
1	*Exercer*		X					
2	*La Revue Prescrire*							
3	*Family Practice*	X						
4	*British Medical Journal (BMJ)*			X		X	X	X
5	*Pédagogie Médicale*							
6	*British Journal of General Practice*	X						
7	*Médecine*							
8	*European Journal of General Practice*	X						
9	*La Revue du Praticien–Médecine Générale*							
10	Cochrane Database of Systematic Reviews						X	
11	*BMC Family Practice*	X						
12	*Annals of Family Medicine*	X						
13	*Revue d'Épidémiologie et de Santé Publique*							
14	*Canadian Family Physician*	X						
15	*Revue Médicale Suisse*							

JMLA Journal of the Medical Library Association

Supplemental content to
J Med Libr Assoc. Apr;106(2):dx.doi.org/10.5195/jmla.2018.282
www.jmla.mlanet.org
© Barreau, Bouton, Renard, Fournier 2018

	Journals	WONCA [1]			Brandon/Hill (2003) [2]		McKibbon (2007) [3]	Alper (2004) [4]
		General practice (English)	General practice (non-English)	General/Internal medicine	General practice	General/Internal medicine		
16	*Journal of the American Medical Association (JAMA)*			X		X	X	X
17	*La Presse Médicale*							
18	*New England Journal of Medicine*			X		X	X	X
19	*Primary Care*							
20	*Concours Médical*							
21	*Minerva Medica*							
22	*Pratiques, les Cahiers de la Médecine Utopique*							
23	*The Lancet*			X		X		X
24	*NPJ Primary Care Respiratory Medicine*							
25	*Scandinavian Journal of Primary Health Care*	X						
26	*American Family Physician*	X			X			X
27	*La Revue du Praticien*							
28	*Le Généraliste*							
29	*JAMA Internal Medicine*							X
30	*Journal of the American Board of Family Medicine*							X
31	*Médecine et Enfance*							
32	*Journal of Family Practice*	X			X		X	X
33	*Preventive Medicine*							
34	*BMC Medicine*							
35	*PLOS Medicine*							
36	*Primary Care Diabetes*							

JMLA Journal of the Medical Library Association

Supplemental content to
J Med Libr Assoc. Apr;106(2):dx.doi.org/10.5195/jmla.2018.282
www.jmla.mlanet.org
© Barreau, Bouton, Renard, Fournier 2018

		WONCA [1]			Brandon/Hill (2003) [2]			
	Journals	General practice (English)	General practice (non-English)	General/Internal medicine	General practice	General/Internal medicine	McKibbon (2007) [3]	Alper (2004) [4]
37	*Swiss Medical Forum–Forum Médical Suisse*							
38	*Canadian Medical Association Journal*			X		X	X	X
39	*Le Médecin du Québec*		X					
40	*Revue Médicale de Liège*							
41	*Australian Family Physician*	X						
42	*La Revue de Médecine Interne*							
43	*Sciences Sociales et Santé*							
44	*Patient Education and Counseling*							

REFERENCES

1. World Organization of National Colleges, Academies and Academic Associations of General Practitioners/Family Physicians. Journals of interest [Internet]. The Association [cited 9 Oct 2017]. <http://www.globalfamilydoctor.com/Resources/JournalsOfInterest.aspx>.
2. Hill DR, Stickel H, Crow SJ. Brandon/Hill selected lists. New York, NY: Gustave L. and Janet W. Levy Library Mount Sinai School of Medicine of New York University.
3. McKibbon KA, Haynes RB, McKinlay RJ, Lokker C. Which journals do primary care physicians and specialists access from an online service? J Med Libr Assoc. 2007 Jul;95(3):246–54. DOI: http://dx.doi.org/10.3163/1536-5050.95.3.246.
4. Alper BS, Hand JA, Elliott SG, Kinkade S, Hauan MJ, Onion DK, Sklar BM. How much effort is needed to keep up with the literature relevant for primary care? J Med Libr Assoc. 2004 Oct;92(4):429–37.
